# Acute gastric dilatation due to a superior mesenteric artery syndrome: an autopsy case

**DOI:** 10.1186/1471-230X-14-37

**Published:** 2014-02-20

**Authors:** Hiroaki Sato, Toshiko Tanaka

**Affiliations:** 1 Department of Forensic Medicine, School of Medicine, University of Occupational and Environmental Health, Japan, Iseigaoka1-1, Yahata-Nishi, Kitakyushu 807-8555, Japan

**Keywords:** Superior mesenteric artery, Duodenum, Mesentery, Scar, Food mass

## Abstract

**Background:**

Superior mesenteric artery (SMA) syndrome occurs when the third portion of duodenum becomes tightly compressed between the SMA and the abdominal aorta (AA). Several causes of the SMA syndrome have been postulated such as marked weight loss, external compression of the abdomen, anatomic variation, and surgical alterations of anatomy. This is an autopsy case of a subject with atypical duodenal obstruction related SMA syndrome.

**Case presentation:**

A 71-year-old woman died one and a half days after eating a large meal of roast meat and vegetables and experiencing subsequent nausea and abdominal pain. At autopsy, fatal acute gastric dilatation was confirmed. The posterior parietal peritoneum around the duodenum was scarred and pulled the root of the mesentery involving the SMA. The complex compressed and narrowed the third portion of the duodenum. The root of the mesentery was also thickened and had adhered to the surface of the duodenum, which may have been due to past peritonitis and disturbance of duodenal motility. Aggregation of an excessively large food mass obstructed the lumen of the duodenum. The cause of death was diagnosed as SMA syndrome with intra-duodenal aggregation of an excessively large mass of food in the narrowed duodenal lumen.

**Conclusion:**

This is an atypical fatal case of acute gastric dilatation, through an excessively large mass of food obstruction at the latent narrowed duodenum.

## Background

Acute gastric dilatation may result in several complications such as dehydration, metabolic alkalosis, gastric necrosis, and systemic circulatory failure**,** all of which are life-threatening [1-5]. Superior mesenteric artery (SMA) syndrome, as well as other predisposing conditions, can cause acute gastric dilatation [6]. In SMA syndrome, the third portion of the duodenum becomes tightly compressed between the SMA and the abdominal aorta (AA). Marked weight loss, external compression of the abdomen, anatomic variation, and surgical alterations of anatomy have been described as causative factors in SMA syndrome [7-10]. The present study describes the autopsy of a patient, with no history of abdominal surgery, who had died from SMA syndrome, the apparent cause of which was atypical acute gastric dilatation resulting from duodenal obstruction.

## Case presentation

The case was a 71 year old female who had no history of abdominal disorders or abdominal surgeries. She had consumed a large meal of roast meat and vegetables for dinner, and soon after experienced nausea and abdominal pain. She visited a physician and underwent a medical examination on the next day. She was diagnosed with gastritis and was prescribed some medications prior to discharge. Two days after the meal, she was found dead lying on her back at home. A large amount of dark brown vomit was found on the floor around her.

**Figure 1:**
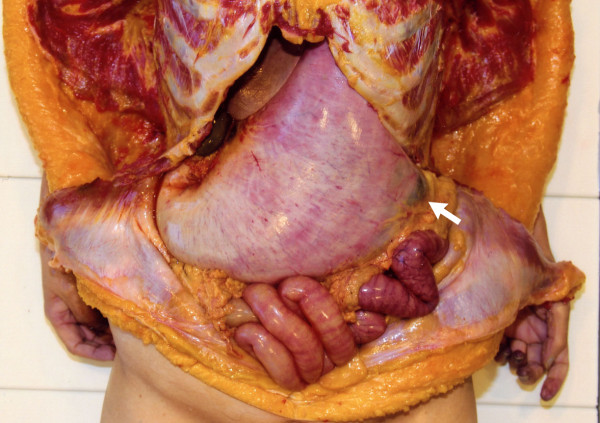
**The opened peritoneal cavity is shown.** A markedly dilated stomach occupied a large part of the abdominal cavity. The surface of the whole stomach showed pallor, and the left proximal part of the greater curvature was gray colored (indicated by arrow), which suggested the possibility of gastric necrosis.

**Figure 2:**
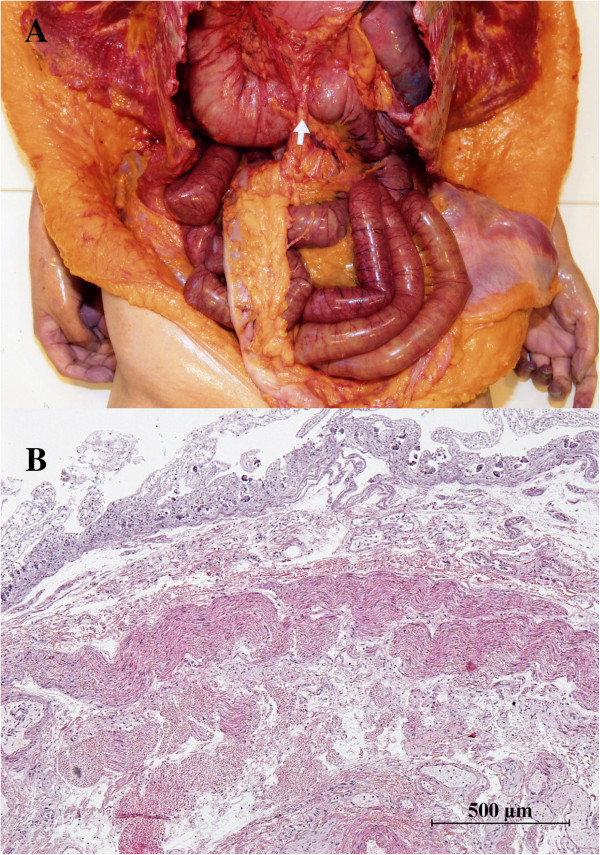
**Lesions of the duodenum, the posterior parietal peritoneum, and the root of the mesentery involving the SMA are shown. A**: The dense fibrotic and thickened peritoneum pulled the root of the mesentery involving the SMA superiorly, which made the SMA tense tightly. The SMA compressed the third portion of the duodenum indicated by small white arrows. In addition, the root of the mesentery was thickened and had adhered to the surface of duodenum, which reduced the duodenal motility. The duodenum proximal to the compressed site was also dilated. **B**: Histopathology of lesion site in the duodenum (hematoxylin and eosin staining). Mucosal necrosis was apparent at the dilated proximal duodenum and there was dense fibrotic degeneration in the thickened peritoneum around the duodenum.

The victim was well nourished, 152.5 cm in height, 48.5 kg in weight, and had a body mass index of 20.8 kg/m^2^. Anatomic deformities or surgical scars were not observed in her chest and abdomen. No vomitus was observed in the larynx, trachea, bronchi, or alveolus. Asphyxia by vomitus was ruled out as the cause of death. Her abdomen was found to be distended. When the peritoneal cavity was opened, a markedly expanded stomach and dilated proximal duodenum were found to occupy a large part of the abdominal cavity. The surface of the stomach showed pallor and the left proximal part of the greater curvature was gray colored (indicated by an arrow in Figure 1), which was suggestive of gastric necrosis, and the mucosal necrosis was apparent at the dilated proximal duodenum (Figure 2B). A dense fibrotic and thickened degenerated area arose from the posterior parietal peritoneum around the duodenum and stretched to the root of the mesentery. The peritoneum was scarred and pulled the root of the mesentery. Dense fibrotic degeneration was found in the thickened peritoneum around the duodenum (Figure 2B). No pathological abnormalities such as arterial thrombosis, arteriosclerosis, aneurysm, or arteritis were observed at the SMA and celiac artery. The root involved the SMA superiorly, made the SMA tense tightly, and resulted in compression and narrowing of the third portion of the duodenal lumen (Figure 2A). The root of the mesentery was also thickened and adhered to the surface of the duodenum, which restricted duodenal motility. The stomach contents consisted of approximately 2 Kg of dark brown fluid containing a large amount of undigested meat and vegetables. An excessively large mass of undigested food was also found in the dilated duodenal lumen, and it had completely obstructed the compressed and narrowed region of the duodenum. We surmised that the women died from acute gastric dilatation accompanied by duodenal obstruction due to SMA syndrome with intra-duodenal aggregation of an excessively large undigested food mass.

## Conclusion

Acute gastric dilatation raises the intragastric pressure resulting in vascular insufficiency [6]. The pressure within the dilated stomach lumen must be greater than 20 cm H_2_O, which exceeds the gastric venous pressure, before mucosal ischemia will occur [6]. The gastric circulatory deterioration causes fragility of the gastric wall, resulting in life-threatening complications such as dehydration, metabolic alkalosis, and mucosal necrosis [2-5]. In addition, acute gastric dilatation increases intra-abdominal pressure and leads to systemic circulatory failure following collapse of the inferior vena cava [1]. The medical literature confirms gastrointestinal necrosis and intra-abdominal high pressure from marked distention of the stomach as features in lethal cases of SMA syndrome [11]. In the present case, the significant dilatation of the stomach (shown in Figure 1) indicated a large increase in abdominal pressure, which could have induced systemic circulatory failure. Taking these findings into consideration, we surmised that acute gastric dilatation was the cause of death in the present case.

For the cause of the gastric dilation, intestinal occlusion by the SMA at the third portion of the duodenum was suspected. The SMA originates from the anterior surface of the AA behind the neck of the pancreas at the level of the first lumbar vertebra. The SMA runs into the root of the mesentery, crosses over the frontal surface of the third portion of the duodenum, and spreads over the intestines. This portion of the duodenum is located between the SMA anteriorly and the AA posteriorly. Imaging studies have shown that aortomesenteric distance between the SMA and the AA was 10 to 28 mm at the sagital plane of the third portion of the duodenum [12]. Reduction in the aortomesenteric distance results in compression of the duodenum, and is a defining characteristic of SMA syndrome [9,10]. The SMA is encased in mesenteric fat and lymphatic tissue at the root of the mesentery, which is important for maintaining a wide aortomesenteric distance under normal conditions. These tissues protect the duodenum from being compressed between the SMA and the AA [9,10]. The most common cause of SMA syndrome is the loss of the fatty padding at the mesentery and the retroperitoneum, which reduces the aortomesenteric distance in debilitating conditions associated with marked weight loss, such as anorexia nervosa, malabsorbtion, and hypercatabolic states [8-10]. In the present case, the third portion of the duodenum was compressed and narrowed severely between the SMA and the AA, but both the mesentery and the retroperitoneum were still protected by an abundance of fat tissue. Some of the factors that have been reported to precipitate SMA syndrome are as follows: external compression of the abdomen induced by wearing tight belts, body spica, or body cast; anatomic anomalies such as lumbar lordosis [7] and high insertion of the ligament of Treitz; and surgical alterations of anatomy such as spine surgery and ileoanal pouch anastomosis [7,9,10]. None of these factors were identified in the present case, whereas a dense fibrotic and thickened degenerated area arose from the posterior parietal peritoneum around the duodenum (Figure 2B) and stretched to the root of the mesentery where the SMA was involved (Figure 2A). The scarred posterior parietal peritoneum pulled the root of the mesentery together with the involved SMA, which made the SMA tense tightly (Figure 2A). These pathological degenerations could have caused a subsequent decrease in the aortomesenteric distance. Several reports have described fatal cases of acute gastric dilatation due to eating a massive volume of food [1,11]. In this case, the root of the mesentery was thickened and adhered to the duodenum, which reduced duodenal diameter. We concluded that this luminal narrowing caused the aggregation of an excessively large mass of food, complete obstruction of the duodenum, and acute fatal gastric dilation at last.

## Consent

Written informed consent was obtained from the family of the patient for publication of this case report and any accompanying images. A copy of the written consent form is available for review form the Editor of this journal.

## Competing interests

The authors declare that they have no competing interests.

## Authors’ contributions

HS performed the autopsy and TT helped draft the manuscript and prepare the figures. Both authors read and approved the final manuscript.

## Pre-publication history

The pre-publication history for this paper can be accessed here:

http://www.biomedcentral.com/1471-230X/14/37/prepub
